# Screening Hospitalized Pregnant Women and Their Male Partners for Possible Distress: A Comparison of the Clinical Usefulness of Two Screening Measures

**DOI:** 10.3390/bs15060767

**Published:** 2025-06-03

**Authors:** Anna Maria Della Vedova, Chiara Bani, Margherita Capretti, Silvia Lucariello, Rita Simonetti, Serena Pelamatti, Emanuela Beretta

**Affiliations:** 1 Department of Clinical and Experimental Sciences, University of Brescia, Viale Europa, 11, 25123 Brescia, Italy; 2 Obstetric Psychology, Department of Gynecology and Obstetrics Operational Unit 1, Azienda Socio Sanitaria Territoriale degli Spedali Civili di Brescia, Piazzale Spedali Civili, 1, 25123 Brescia, Italy; banichiara1@gmail.com (C.B.); margheritacapretti@gmail.com (M.C.); lucariellosilvia@gmail.com (S.L.); rita.simonetti92@gmail.com (R.S.); emanuela.beretta@asst-spedalicivili.it (E.B.); 3 Department of Mechanical and Industrial Engineering, University of Brescia, Via Branze, 38, 25123 Brescia, Italy; serena.pelamatti@unibs.it

**Keywords:** screening, pregnancy, anxiety, depression, hospitalization, risk

## Abstract

Pregnancy is a period of great complexity and potential psychological vulnerability which may increase in unfavorable conditions, such as hospitalization. Therefore, early identification of emotional, anxious, or depressive difficulties is important in terms of maternal and fetal well-being. International guidelines recommend the use of brief screening tools to identify perinatal women for further investigation, but which of the measures is optimal remains to be clarified. The objective of this study was to compare the Whooley depression questions used together with the Generalized Anxiety Disorder-2 (GAD-2), versus the Matthey Generic Mood Questionnaire (MGMQ), to evaluate their concordance in screening results and their clinical usefulness in the hospital setting. Hospitalized pregnant women, numbering 228, and 55 male partners completed both questionnaires. The women’s results showed high similar screen positive rates on both measures, but the screen positive concordance between the two instruments was low (around 50%). The Whooley/GAD-2 missed a significant percentage of women who, on the MGMQ, wished to talk with a professional, or who expressed moderate to high distress. The data from male partners were too few to be interpretable. Findings suggest that screening is important in hospitalized women, and clinicians should consider the respective merits and possible weaknesses of different screening tools.

## 1. Introduction

Motherhood is a phase of great complexity, and it is recognized that under unfavorable conditions it can lead to potential psychological vulnerability (O’Hara, 2024). The prevalence of probable distress (depression or anxiety) in women during pregnancy is estimated to be around 20% (Falah-Hassani et al., 2017; Fawcett et al., 2019; Woody et al., 2017) with a noteworthy increase in the post-COVID-19 period especially in low-income population (Al-abri et al., 2023; Nielsen-Scott et al., 2022). Furthermore, the risk of experiencing perinatal emotional difficulty may increase under particular unfavorable conditions, such as psychosocial and economic fragility, educational poverty, isolation, health problems, or risky pregnancy (Dagher et al., 2021; Louis-Jacques et al., 2020). To date, the consequences of perinatal maternal distress on the woman’s and fetus health and the newborn’s development have been widely documented (Bauer et al., 2016; Stein et al., 2014), drawing attention to the importance of prevention. The literature also shows that approximately 10% of men report symptoms of perinatal depression or anxiety (Glasser & Lerner-Geva, 2019; Leiferman et al., 2021) with important consequences on the well-being of couples and on child development (Gutierrez-Galve et al., 2019).

For all these reasons, the importance of carrying out a screening for maternal emotional distress, anxiety, or depression in the perinatal period is now shared in a large number of countries and is recommended by the National Institute for Health and Care Excellence (NICE) guidelines (NICE, 2014) and by the American Academy of Pediatrics (Earls et al., 2019). Recently the World Health Organization (2022) (*Guide for integration of perinatal mental health in maternal and child health services*, 2022) emphasized the importance of screening within a stepped-care model, which represents the best approach for perinatal mental health. In some countries, for the same reasons, screening for emotional distress is now starting to be implemented in fathers-to-be and new fathers (Della Vedova et al., 2023a; Walsh et al., 2020).

When considering conditions of specific vulnerability of the woman or the family, being able to identify emotional distress in time becomes even more important. Among the conditions of increased psychological vulnerability is that of an event that requires the hospitalization of the woman during pregnancy. A recent meta-analysis highlighted that in hospitalized pregnant women the percentage of depression and anxiety doubles compared to the general population (Toscano et al., 2021)**,** and a further study found a higher percentage of suicidal thoughts (Hines et al., 2018).

Health problems that require hospitalization can affect the woman’s emotional well-being and cause concerns about the progress of the pregnancy or the health of the unborn child, significantly threatening the sense of continuity in the psychological process of transition to parenthood, as well as hindering the development of the maternal attachment to the fetus (Pisoni et al., 2014; Smorti et al., 2023). An abrupt and unexpected admission to the hospital can increase anxiety levels in pregnant women (Zych-Krekora et al., 2024) and furthermore, the presence of distress can also affect men, who fear possible consequences for the health of their partner and the fetus, and experience their and their children’s distress due to the woman’s absence from home. For all these reasons, carrying out a screening of the emotional distress that hospitalized pregnant women (and their partners) experience can be important so that timely management of perinatal distress is possible (Beretta et al., 2023; Capretti et al., 2022; Masserdotti et al., 2022). Furthermore, this study is grounded on the theoretical framework of psychoanalytic psychology, where becoming a parent constitutes a moment of great biopsychosocial complexity (Della Vedova, 2014; Della Vedova et al., 2023b; Matthey & Della Vedova, 2020). For women this delicate moment of maturational crisis (Bibring, 1959; Bydlowski, 2020), in unfavorable conditions such as hospitalization, can result in specific vulnerability and stress. Therefore, having the possibility of clinical listening in the hospital environment can help in containing a possible emerging psychological difficulty.

### 1.1. What Tools Are Available for Screening in the Perinatal Period and Which One Can Be the Most Suitable in a Hospital Context?

When talking about screening, a broad discussion opens up which inevitably leads to the choice of a specific instrument and the reasons underlying this choice—a very complex issue. A recent literature review on screening tools for perinatal depression and anxiety highlighted that there is a need for systematic research examining which screening tools to use and also a lack of screening for perinatal anxiety (Bhat et al., 2022). A further study examined the effectiveness of validated screening tools, highlighting the complexity and multiple aspects to consider in implementing a screening program (Sambrook Smith et al., 2022). The results of a large systematic review and meta-analysis on the effects of screening programs which employed different screening measures (Waqas et al., 2022), found that it is effective in reducing perinatal depression and anxiety. In recent years, routine universal screening for perinatal anxiety and depression symptoms have been put in place in several countries (e.g., England, Australia, Italy), with international guidelines recommending different measures. The National Institute for Health and Care Excellence (NICE) guidelines (NICE, 2014), currently recommend the two Whooley depression questions (Whooley et al., 1997), together with the GAD-2 anxiety questions (Kroenke et al., 2007; Spitzer et al., 2006), as the first step in perinatal mental health screening. These ultra-short instruments are particularly suitable for very busy primary and secondary care settings, where staff do not have time to administer longer instruments and score them. These screening measures take a symptomatic approach to detecting possible distress, and use a ‘cut-off score’ on each to determine if a woman needs further assessment. However, as is known, investigating specific symptoms with self-report can have advantages and disadvantages: in a short instrument not all symptoms can be investigated, the cut-offs can vary greatly in different populations, and the questions can be misunderstood, thereby generating false positives or negatives (Matthey & Agostini, 2017).

In this regard, recent research (Matthey et al., 2023) has detected several problems with some of the most used brief screening measures. For instance, when completing the Whooley questions, about 15% of English-speaking women completely misinterpret the Whooley question number two (from now on called ‘Anhedonia Question’) which investigates the anhedonia symptom (namely the inability to experience joy or interest due to low mood), and approximately 17% admit that they have difficulty understanding it. It has also been discovered that endorsement of the anhedonia symptom is often due to the normal physical changes in pregnancy, and not due to low mood (about 65%) (Matthey et al., 2023; Matthey & Ross-Hamid, 2011). This means that a significant percentage of women do not understand Whooley’s Anhedonia Question and respond positively, when instead their lack of interest is related to the normal focus on pregnancy, generating false positive screenings.

Furthermore, given that different measures often do not enquire about identical symptoms for the same construct, it is not surprising that concordance between various validated perinatal emotional health screening measures can be quite low (Affonso et al., 2000; Condon & Corkindale, 1997; Hanna et al., 2004). Condon and Corkindale (1997), for example, found that only between 28% and 56% of women who screened positive on one measure, based on depression symptoms and their frequency, also screened positive on another measuring the same construct. This poses a significant problem for clinicians, namely that depending on the tool chosen, some women who may have emotional difficulties may not be identified.

One way to try to overcome these problems might be to use tools that use broader and more clinical in nature questions, rather than a symptomatic approach. This approach is the basis of a different type of emotional health screening measure, the Matthey Generic Mood Questionnaire (MGMQ) (Matthey et al., 2013, 2019).

The MGMQ differs from the Whooley questions plus GAD-2 (from now on called Whooley/GAD-2) in that it enquires about the construct of feeling ‘distressed’, rather than asking about specific symptoms. It asks a woman directly how much she is “bothered” by her feelings, and also whether she wishes to talk to a health professional about how she is feeling. It is the answers to these latter two questions that determine if a further assessment is required.

Considering the importance of early identification of possible distress in hospitalized pregnant women, a research project with the aim to evaluate the relative merits of the Whooley/GAD-2 and MGMQ was implemented in the clinical setting of the Obstetrics Department of Spedali Civili di Brescia, a public hospital in a medium-sized city in the Lombardia region in northern Italy. In this hospital, the perinatal psychologist (the last author) and her team as well as the principal investigator of this study (the first author), share a psychodynamic, psychoanalytic vision of parenthood. For this reason, they favor a targeted approach to offering listening and containment to women in addition to a diagnostic investigation in view of treatment where necessary. They are therefore interested in a screening that is more oriented towards the general well-being of the woman rather than a screening based only on a quantification of symptoms.

Thus, the overall objective of the study was to understand the comparative performance of the Whooley/GAD-2 and MGMQ in Italian speaking women hospitalized during their pregnancy to understand which tool has the best clinical usefulness in this setting. Where possible, the women’s partners were also offered the opportunity to participate, in order to assess their level of distress.

### 1.2. Research Questions

The research questions concerned the comparative performances between the Whooley/GAD-2 and the MGMQ for Italian women in this particular clinical setting, with respect to the following: (a) satisfaction with, and preference for, the two measures; (b) interpretation of Whooley’s Anhedonia Question (equivocal or exact); (c) attribution of the anhedonia symptom to low mood or physical changes in pregnancy; (d) screen positive concordance between the two instruments, Whooley/GAD-2 and the MGMQ, in hospitalized women and their partners, respectively.

## 2. Materials and Methods

### 2.1. Procedure

Ethics approval was obtained from the Ethics Committee of the Brescia’s public hospital (Spedali Civili di Brescia). All participating women and men signed the ethic’s approved consent form. Women admitted to the Obstetric Department with pregnancy complications or other medical conditions, from September 2022 to March 2023 were recruited consecutively on the mornings of non-holiday Mondays through to Fridays. Eligible women were pre-selected by the head nurse of the ward if they met the following criteria: Italian speaking; the admission was for pregnancy complications or other medical reasons, but not for an emergency requiring an immediate surgical or similar intervention.

Eligible participants were recruited within 2 days of being admitted to the Obstetrics Department by several different Project Officers (POs), who were research psychologists involved in the project; each woman who had a male partner was asked if she would give permission for the PO to contact him, to see if he would also consent to participate in completing the questionnaires for himself, via telephone or in person. Confidentiality was assured to the woman (and man if he participated) that the answers on the forms would not be communicated to the partner. After completing the questionnaires, women were asked questions by the POs to clarify any responses and to verify their understanding of the questions and their satisfaction with the two measures.

If a woman screened positive on any of the questionnaires (i.e., the Whooley/GAD-2 or MGMQ), she was offered the appropriate professional support routinely given to all inpatients from the psychologist specialist of the obstetrical unit. All measures and communications were in Italian.

### 2.2. Measures

#### 2.2.1. Modified Whooley Questions

The Whooley questions (Whooley et al., 1997) are the two core-depression diagnostic questions in the Structured Clinical Interview for Diagnostic and Statistical Manual of Mental Disorders, which investigate symptoms of low mood and anhedonia, respectively (First, 2015). The original two Whooley questions use a time frame of the past month. This was changed to the ‘past 2 weeks’ for this study, as was done in previous research (Matthey et al., 2023), so as not confuse participants, given that both the GAD-2 and the MGMQ (see below) enquire about the past 2 weeks. Thus, in English the modified Whooley questions are as follows: ‘1. During the past 2 weeks have you often been bothered by feeling down, depressed, or hopeless? (Low Mood Question)’; ‘2. During the past 2 weeks have you often been bothered by having little interest or pleasure in doing things?’ (Anhedonia Question). The response options for each question are ‘Yes’ or ‘No’, as per the original Whooley questions. A response of ‘Yes’ on at least one of the questions is considered to be ‘screen positive’ for possible depression. A sensitivity value of 100% for the diagnosis of depression was found for Whooley questions in a UK perinatal validation study (Mann et al., 2012) and in an American postnatal study (Gjerdingen et al., 2009). Of note is that a recent Chinese validation study (Wu et al., 2024) found a much lower sensitivity of 77% and a very high proportion of false positives (positive predictive value of only 0.2).

#### 2.2.2. GAD-2

The Generalized Anxiety Disorder-2 (GAD-2) is the short version of Generalized Anxiety Disorder-7 (Spitzer et al., 2006) and it comprises the following two questions regarding anxiety. ‘Over the last 2 weeks, how often have you been bothered by the following problems?: 1. Feeling nervous, anxious or on edge; 2. Not being able to stop or control worrying’. The response options for each question (with their respective score in brackets) are the following: ‘Not at all (0); Several days (1); More than half the days (2); Nearly every day’ (3). A total score of 3 or more across the two questions is considered to be screen positive.

The GAD-2 performs well in the general population (Kroenke et al., 2007), but there are not many perinatal validation studies. One study highlighted some limitations of GAD-2 in generating many false positives (Nath et al., 2018)**,** and a more recent study (Ayers et al., 2024) found in a perinatal English-speaking sample that a score of 2 or more performed better than the usually recommended 3 or more.

#### 2.2.3. MGMQ

The MGMQ (Matthey et al., 2013, 2019) consists of four questions: Q1. A distress question (with a time frame of during the past 2 weeks)—‘Have you felt very stressed, anxious, or unhappy, or found it difficult to cope, for some of the time?’ (response options: ‘Yes’, ‘Possibly’, ‘No’); Q2. A bother impact question (for those who answered ‘Yes’ or ‘Possibly’ to the distress question)—‘How bothered have you been by these feelings?’ (response options: ‘Not at all’; ‘A little bit’; ‘Moderately’; ‘A lot’); Q3. A reason for distress question, asking the individual why she is feeling this way (if appropriate, and only if she wishes to describe this on the questionnaire); Q4. A wish for referral question—‘Would you like to talk to a health professional about any of these things?’ (response options: ‘Yes’, ‘Possibly’, ‘No’).

This measure has performed well for women in antenatal and postnatal clinical settings against diagnostic criteria for depression and anxiety and against various self-report measures of anxiety and depression, for English-speaking women (Matthey et al., 2013, 2019; Matthey & Bilbao, 2018; Price et al., 2021), for Italian-speaking women and men (Matthey & Della Vedova, 2018, 2020), and also Arabic-speaking postpartum women (Matthey et al., 2024). Screen positive responses on the MGMQ are as follows:

(i) Moderate/Major distress: an answer to question Q2 (Bother Impact question) equal to: ‘Moderately (bothered)’ or ‘A lot (bothered)’; and/or on Q4 (Wish to talk question): ‘Yes (I want to talk with a health professional about how I am feeling)’.

(ii) Minor distress: an answer to question Q2 equal to: ‘A little bit (bothered)’, together with on Q4: ‘Possibly (I want to talk with a health professional about how I am feeling)’.

The Moderate/Major distress classification was determined by consulting with Italian clinicians (Matthey & Della Vedova, 2018), as well as the measure’s performance against diagnostic criteria (Matthey et al., 2019), while the Minor distress classification was determined by clinical considerations.

#### 2.2.4. Women’s Views, Understanding, and Interpretation of the Measures

A series of questions was developed to understand what the women thought of the different measures. Some of these questions were partly derived from previous research on these issues (cf., Matthey et al., 2023; Matthey & Ross-Hamid, 2011). The questions explored the following: (i) the women’s opinions of each measure, and whether they had a preference for one or the other; (ii) whether or not they had correctly understood the Whooley Anhedonia Question, and the MGMQ Bother Impact question (MGMQ Q2); (iii) whether they attributed the anhedonia symptom (if applicable) to their mood/worries, or to the usual physical symptoms and changes of being pregnant; (iv) whether on the MGMQ Q2 and Q4 they preferred the current response options, which includes the option of saying ‘Possibly’, or whether they considered it would be better to just have the options of ‘Yes’ and ‘No’. Men were not asked these questions due to the practical limitation of not having face-to-face contact with most of these participants.

### 2.3. Sample Size

To report on the percentage of women screening positive on each measure, with an accuracy of +/−5% at the 90% confidence level, with a screen positive rate of at least 30% (given that this was a ‘high-risk sample of women, and thus would be expected to have a higher screen positive rate than a normal community sample), a sample size of 229 was required (Calculator.net, n.d.). This sample size was almost completely achieved for the women (n = 228), but not, as was anticipated, for the male partners. The rates and other data reported for the men, therefore, should be treated very cautiously and are reported to give an initial view of the distress of partners of hospitalized women.

### 2.4. Statistical Analyses and Effect Size

Data were analyzed in terms of frequences and proportions. The chi-square test for homogeneity was used to evaluate whether the proportion of similar positive case rates across both measures differs significantly (a *p*-value less than 0.05 suggests that the null hypothesis of homogeneity can be rejected). Cohen’s kappa was used to assess the concordance between the screening tools considering that a k ≤ 0.20 means a poor strength of agreement, k = 0.21–0.40 fair, k = 0.41–0.60 moderate, k = 0.61–0.80 good, k = 0.81–1.00 a very good agreement (Landis & Koch, 1977). McNemar’s test was used to check for asymmetry in discordant cases. Furthermore, as has been reported in similar research (Matthey et al., 2023), to ascertain clinically meaningful differences in the analyses, the effect size was used. As proposed by Rosenthal (Rosenthal, 1996) the ‘percentage point difference’ method suggests that when the smaller percent is between 7% and 15%, a medium effect size is 12 percentage points difference, and a large effect size is 20 percentage points difference. When the smaller percentage is between 15% and 85%, a difference of 7 percentage points is a small effect size, 18 percentage points is medium, 30 percentage points is large, and about 45 percentage points is a very large effect size. The analysis was conducted with SPSS Statistics, V.23 (IBM Corp., 2015, Armonk, NY, USA).

## 3. Results

### 3.1. Participants

**Figure 1:**
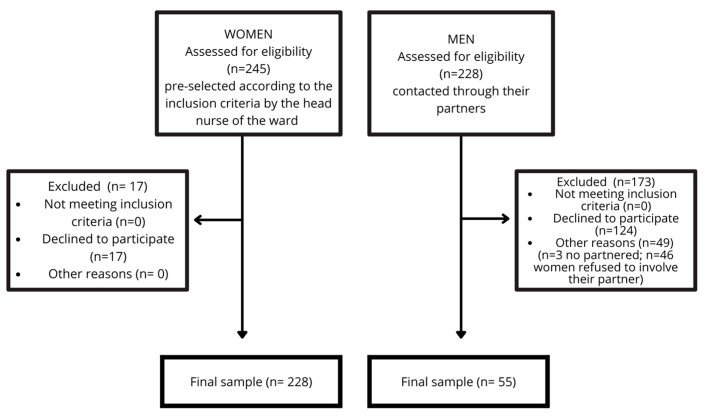
CONSORT-style diagram of sample information.

The characteristics of the sample are reported in Figure 1.

### 3.2. Demographics

The sample was composed of Italian women and some of their partners; women were mostly partnered, highly educated, primiparous, and in the third trimester of gestation. The reasons for hospitalization were mostly related to pregnancy problems (52%—e.g., increased blood pressure, preeclampsia), general health reasons (23%—e.g., minor accidents, renal colic), risk of pre-term birth (20%—e.g., pre-term contractions, shortening of the uterine cervix), with the remaining 5% having missing data. The demographic information for the participants is displayed in Table 1.

### 3.3. Rates of Positive Screening (MGMQ and Whooley/GAD-2) with Respect to Women’s Sociodemographic and Pregnancy Variables

Rates of positive screening in women were 35.5% on the Whooley/GAD-2 and 33.9% on the MGMQ, while in men they were 20% on the Whooley/GAD-2 and 15.1% on the MGMQ. Considering the women’s sample, the chi-square statistic showed that no sociodemographic variable (age, education, parity, nationality) or the partner’s participation in the study was significantly associated with positive screening results for MGMQ or Whooley/GAD-2. There was a small-moderate greater percentage of women with mild or moderate-major distress in the women admitted for pre-term birth reasons (46%), compared to those admitted for pregnancy complication reasons (31%) and those admitted for general hospitalization reasons (29%). The percentage point differences was of 15 and 17, respectively, thus a small-moderate effect size.

### 3.4. Women: Perception and Understanding of the Measures

As shown in Table 2, the rate of satisfaction for each instrument was high, and there was a greater proportion of women preferring the MGMQ over the Whooley/GAD-2 (50% vs. 37%—13 percentage points difference, small-to-medium effect size).

The reasons why women said they preferred one or other of the measures were similar. Some women said that they preferred the MGMQ because of its open question (Q3), where they could express how they felt, whereas others said the opposite—that they preferred the Whooley/GAD-2 because it did not have such an open question, and therefore it is quicker to respond. Similarly, some preferred the MGMQ, saying that it was easier to understand, while others felt that the Whooley/GAD-2 was easier to understand.

There was a substantially stronger preference for the inclusion of the response option of ‘Possibly’ in both the MGMQ Distress (Q1) and Wish-to-talk (Q4) questions (both with 30 or more percentage points difference—thus, a large effect size). The reasons included that some women may not wish to admit saying ‘Yes’ to such questions, and thus having ‘Possibly’ gave them an easier way of admitting that they felt distressed, or wanted to talk to someone. Others said that ‘Possibly’ was a good option as some women may be unsure of exactly how they are feeling (Q1), or whether or not they wanted to talk to someone (Q4).

Of note is that four women completely misinterpreted the Whooley Anhedonia Question (saying ‘No’ to this question, but in fact they meant to say ‘Yes’ when the question was checked by the PO), and one found it confusing. No women, however, misinterpreted the MGMQ Distress Impact question (Q2). Of those who endorsed the Whooley anhedonia symptom (N = 17), around half (n = 8) said that it was just due to the physical symptoms of pregnancy and not due to low mood or worries. For example, some said that having to stay at home and rest, due to their pregnancy complication, meant they could not enjoy things like they used to.

### 3.5. Women: Wish-to-Talk to a Health Professional and Their Reasons (MGMQ Q3 and Q4)

Table 3 shows that 23.7% of the women either said ‘Yes’, or ‘Possibly’, to the Q4, Wish-to-Talk, question. Their reasons included the following: (i) Problems or concerns with their pregnancy (e.g., feeling very worried that something may happen to their infant; concern about the possibility of an abortion, having previously experienced this); (ii) Concern that they may not have enough support, or may not cope with everything, once the baby is born (e.g., due to moving house, having to work, and the mother’s own health); (iii) Interpersonal difficulties or stresses (e.g., concerns regarding the relationship with her partner, her own mother’s poor health). There is however no clear pattern as to the reasons a woman wants to, or does not want to, talk with a health professional. This will probably depend upon a variety of factors, including the level of distress she is experiencing, her own coping style, her support systems, and her previous experience at receiving help.

Of note is that on the MGMQ, the wish to talk with a health professional (Q4) was not always related to the degree of ‘bother’ a woman reported on the MGMQ’s Q2. Thus, 7 of the 16 women saying ‘Yes’ they wanted to talk with someone said that they were only ‘a little bit’ bothered by how they were feeling (Q2). This was also evident for those saying they ‘Possibly’ wanted to talk with a health professional (16 of the 37 women were only bothered ‘a little bit’).

Furthermore, for women being positive on the Impact question of the MGMQ (Q2: saying that they were bothered either ‘Moderately’ or ‘A Lot’: N = 54), this did not necessarily mean that they wanted to talk with a health professional. Of these 54 women, 27 (half) said that they did not want to talk with a health professional.

### 3.6. Men: Wish-to-Talk to a Health Professional and Their Reasons (MGMQ Q3 and Q4)

Table 3 shows that a much smaller percentage of men, compared to women, said that they wanted to talk with a health professional (0% ‘Yes’; 3.8% ‘Possibly’). The reasons these two men (3.8%) gave for feeling ‘distressed’ included one having to cope with work, moving to a new house, and the pregnancy, without wanting to burden his partner with these stressors; and the other man’s partner had experienced a misdiagnosis requiring admittance to hospital and he had difficulty visiting her in hospital.

Other reasons for men’s ‘distress’ (‘a little bit’ or ‘moderately’ bothered on MGMQ Q2), despite not wishing to talk with a health professional, were related to the following: pressures of work; concern for his partner’s health; concern for his partner who is at home alone (referring in general to the times when he could not be near his partner because he was at work); needing to do many more things at home due to his partner’s poor health; lack of sleep due to the impending birth; health problems with their relatives.

As with the women, however, it is noteworthy that one of these two men that ‘Possibly’ wanted to talk with a health professional (MGMQ Q4) said that he felt distressed just ‘a little bit’ (Q2). Therefore, despite this much smaller sample size in men, it is also possible that the level of bother and the (possible) wish to talk with a health professional may not be strongly related. In addition, all six men who said that they were bothered ‘Moderately’ (none said ‘a lot’), said that they did not want to talk with a health professional.

### 3.7. Comparison of the Two Instruments: Women

Rates of screening positive on the two measures were similar for the women (about 35%—see Table 3). However, the concordance for screen positive between the two instruments was only ‘average’, with about a third of the women screening positive on one instrument then screening negative on the other instrument (see Table 4). The chi-square value was χ^2^ = 62.29 (*p* < 0.001, Phi = 0.52) indicating strong evidence against the null hypothesis of homogeneity between the two questionnaires. Cohen’s concordance index (κ) was equal to 0.496, indicating a moderate level of concordance between the two instruments according to the commonly used interpretative thresholds. This suggests that, although there is a certain coherence between the answers provided in the two questionnaires, there are also non-negligible discrepancies. McNemar’s test was also applied to check for asymmetry in discordant cases. The observed values (24 subjects positive only in the MGMQ, 28 subjects positive only in the Whooley/GAD-2 test) did not show a statistically significant difference (χ^2^ = 0.173; *p* > 0.05), indicating that the discrepancies between the two questionnaires do not follow a systematic direction.

Furthermore two of the 17 women (ca., 12%) who said on the MGMQ that that they wanted to speak with a health professional (responding ‘Yes’ to Q4) were negative on the Whooley/GAD-2, and 15 of the 37 (41%) who said that they ‘Possibly’ wanted to talk with a health professional were also negative on the Whooley/GAD-2. Thus, 17 of the 54 women (31%) who either said ‘Yes’ or ‘Possibly’ I want to speak with a health professional on the MGMQ Q4 were ‘missed’ (negative) by the Whooley/GAD-2.

Of the 28 women who were positive on the Whooley/GAD-2 but were negative (and thus ‘missed) by the MGMQ, all therefore had said on the MGMQ that they did not wish to talk with a health professional, and also that they did not consider their distress to be at the level of ‘Moderately’ or ‘a lot’.

### 3.8. Comparison of the Two Instruments: Men

Considering that the number of men who agreed to participate in the research was very low, we report the results to open an initial view on paternal distress in conditions of hospitalization of the pregnant woman. The rates of screen-positive for men between the two instruments were similar (15–20%; see Table 3). Among the eight men positive on the MGMQ, only one was negative on the Whooley/GAD-2. This man was ‘Moderately’ bothered by how he was feeling, saying on Q3 “the fact that my wife isn’t well”, though also stating that he did not want to talk with a health professional.

Of the 11 men positive on the Whooley/GAD-2, seven were positive on the MGMQ, but four were negative. For these four men, while on the Whooley, they said ‘Yes’ to at least one of the two questions (n = 3), or had a score of 3 or more on the GAD (n = 1), on the MGMQ Q1 they either said that they were not distressed at all (n = 1), or on Q2 said that they were only bothered a ‘little bit’, and did not want to talk with a health professional (n = 3). The reasons the three men gave for feeling just a little bit bothered, and not wanting to talk to a health professional, were as follows: “my partner is home alone”; “too little sleep and the approaching birth”; “our other child cannot see his mother as she’s in hospital”.

## 4. Discussion

The aim of this study was to evaluate the clinical usefulness of different screening tools in the setting of pregnancy hospitalization, considering international guidelines recommendation (NICE, 2014) and the preventive value of screening for perinatal mental health (*Guide for integration of perinatal mental health in maternal and child health services*, 2022), within a psychodynamic framework of the complexity of the psychological processes of parenting. The results highlight that the percentage screening positive on emotional measures for hospitalized women are higher than in community samples as shown by international literature (Toscano et al., 2021) and as would be expected, given that the pregnant women had been admitted to hospital with health complications. In agreement with the literature, this result confirms the distress burden exerted by the condition of greater risk of hospitalized women, especially in cases of risk of pre-term birth, and reiterates the importance of screening for perinatal distress in hospital settings (Smorti et al., 2023). Findings from the current study also suggests that partners of hospitalized women may suffer clinically relevant distress.

Regarding the appreciation by women of the instruments, both the Whooley/GAD-2 and the MGMQ were considered acceptable by the Italian-speaking women in this sample. A greater proportion of women preferred the MGMQ over the Whooley/GAD-2 (50% vs. 37%), with a large majority preferring the inclusion of the ‘Possibly’ response option in both MGMQ Questions, Q1 (67% vs. 32%) and Q4 (71% vs. 29%), these differences being large effect sizes and thus clinically meaningful. Of note is that the Whooley does not provide this response option, only those of ‘Yes’ and ‘No’.

With respect to the clarity of the questions perceived by women, in particular the understanding of the Whooley Anhedonia Question, as has been found in previous research (Matthey et al., 2023; Matthey & Ross-Hamid, 2011), a sizeable proportion of women (half) endorsing the anhedonia symptom on the Whooley said it was only related to the normal physical changes of being pregnant, and was not due to their mood or worries. Furthermore, a few completely misinterpreted the Whooley Anhedonia Question (n = 4). This percentage is much smaller than found in the English-speaking study (Matthey et al., 2023) and would suggest that the Italian wording for a loss of interest and pleasure is not ambiguous, but that the misinterpretation might be due to the skim reading phenomenon, as suggested by Matthey et al. (Matthey et al., 2023). Of note is that Bruney and Zhang (Bruney & Zhang, 2022) also reported that around 10% of their screen-positive Patient Health Questionnaire-2 (PHQ-2) sample, who were interviewed, had completely misinterpreted one or other PHQ-2 question (which are the same as the Whooley questions). Thus, it seems to emerge clearly from these data, that, without explicitly exploring their comprehension of the Anhedonia Question, the use of the Whooley as a screening measure in Italian women seems to be as problematic as it is in English-speaking women.

Considering the main objective of the study, the concordance between the Whooley/GAD-2 and the MGMQ, the results of the study show that while screen positive rates on both measures were similar (both for women, and also for men), the agreement was moderate, highlighting non-negligible discrepancies. In particular, around a third of the women who screened positive on one measure screened negative on the other. This means that by using one measure or another some women will be missed (will screen negative).

One may wonder what this means in different cases, and whether this screen positive concordance percentage is acceptable, especially in a condition of increased distress, such as hospitalization. Even if this result replicates the finding from previous research using other perinatal mental screening measures (Condon & Corkindale, 1997), we believe that this lack of screen positive concordance between measures should be a major concern for researchers and clinicians in this field.

An important point in the comparison between the two instruments concerned the desire of women to talk to a professional about their emotional difficulties (Q4 MGMQ). The data show that 17 women (31%) who said they would like, or would possibly like, to talk to a professional, screened negative on the Whooley/GAD-2. This means that in a service using just the Whooley/GAD-2, these women’s wishes would not have been identified.

We would argue that asking a woman directly how distressed she feels and whether or not she wishes to talk with a health professional should take priority over a numerical score based upon the frequency of symptoms, where the assumption is that a certain level of frequency warrants intervention. To decide, for example, that a woman who says that she wants to talk with a health professional should not receive any such service because she only scored 2 on the GAD-2, or because she said ‘No’ to both of the Whooley questions, seems to be lacking the human element of listening to what the woman is really experiencing. While guidelines often state that further clinical judgement should always be used to determine if further assessment is required (Highet, 2023), rather than just rely on a woman’s ‘score’ or responses to such screening questions, the reality is that few services have the time or resources to provide skilled in-depth questioning to every woman who is screened. Furthermore, in this sample, women’s wish to talk with a health professional was not necessarily linked to the intensity of their distress. This reveals that even women who declare mild distress may wish to talk to an operator, something that could not be detected by a cut-off or a self-report questionnaire. On the other hand, a considerable number of women with high distress did not express the wish to talk with a health professional, and this also reveals that the number of women who could turn to referral services would not be many, and therefore not overwhelm the service resources even in the case of universal screening.

The use of a screening measure like the MGMQ minimizes the risk of missing women who do indeed have a substantial level of distress, or who wish to talk with a health professional regardless of the frequency of specific symptoms they are experiencing. This may be especially important in the inpatient hospital setting, because there are not always routine scheduled visits as in outpatient perinatal services. A tool that immediately provides clinically relevant information is therefore very useful to open a conversation and possibly propose a referral.

Another interesting aspect concerns the reasons that women reported for their distress. In line with other Italian studies, they were similar to the typical concerns of motherhood but with greater emphasis on fears for the pregnancy outcome, the health of the woman and the child, and the distress due the woman’s absence from home (Smorti et al., 2023). The reasons given by men are very similar and emphasize concerns about the health of their partner and the fetus as well as the difficulty in managing work and household matters in the absence of the woman.

In the current study, carried out in an obstetric hospital department where screening has been implemented for several years (Beretta et al., 2023; Capretti et al., 2022; Masserdotti et al., 2022), the use of the MGMQ’s brief questions enabled the clinicians (and researchers) to understand some important clinical issues and provided an opening for the clinician to talk to the woman about how she was feeling. This represented very useful information for planning a referral to the Obstetric Unit’s Psychologist.

More in general, we may argue that the results of the study enabled us to identify the characteristics that make an instrument the most suitable based on the context of the hospitalized women. In the perinatal period, and especially in situations of significant maternal concern, the use of screening tools that open the possibility for dialogue about the causes of potential emotional distress and the possibility of requesting to speak with healthcare providers could be helpful, as seems to emerge from this study. Overall, from this study several aspects emerge that could indicate the need to reconsider the limitations of screening measures based only on the intensity and frequency of certain symptoms, and it may suggest a reflection on areas for improvement in the practice guidelines for mental health care in postpartum care.

Moreover, in the specific theoretical psychodynamic, psychoanalytic framework of parenting on which this research is based, a tool of this type meets the importance of offering empathic listening to the concerns of pregnant women and their partners in order to offer sensitive and targeted counseling to the specific needs of this population.

The data also show that men (even if they agreed to participate in a small percentage) showed willingness to fill out the MGMQ questions and even the one about wishing to talk with a health professional. This confirms a previous finding when using the MGMQ with men in Italy (Matthey & Della Vedova, 2020) and other studies in which the extension of perinatal distress screening to men was found to be desirable (Walsh et al., 2020) and welcomed (Della Vedova et al., 2023a).

### Limitations of the Study

A limitation of the study could be the recruitment of women, which could only take place on working days. Furthermore, the composition of the women’s sample (as women were mostly Italian, partnered, highly educated, and in the third trimester of gestation) represents a limit to the generalizability of the results and application to other groups. Therefore, future research may explore the clinical usefulness of these screening tools in larger, more heterogeneous samples. In addition, the screening measures we used have no diagnostic value, and only women who tested positive had a subsequent assessment. A further strong limitation was the low uptake rate and thus the small sample size of the men. Therefore, for the male sample the results should be taken with caution. Finally, since we did not investigate gender identity, we refer to the participants as women, but we recognize that some of the patients may not identify as women, the same applies to men.

## 5. Conclusions

This study, conducted in a hospital clinical setting and within a psychodynamic framework of psychology of parenthood, shows the importance of performing an accurate screening of emotional distress in perinatal women who experience hospitalization. It also shows that screening their partners, when possible, can be important too. However, it highlights some aspects related to the performance of screening tools that deserve attention. The results show that there are differences between the screening symptom questionnaires of the Whooley questions and GAD-2 and the construct questionnaire of the MGMQ. This latter measure appears to have the advantage of asking women directly how they feel, why they might be feeling this way, and whether they would like to talk with a health professional. Furthermore, many of the women and some of the men, who were ‘screen positive’ on the MGMQ and who said that they wished to, or possibly wished to, talk with a health professional were missed when screening by using the Whooley questions along with the GAD-2. Thus, the results of this study may help clinicians to be aware of the respective merits and possible weaknesses of different screening tools, to enable them to select a suitable instrument for their clinical practice. Additionally, the results of the study may suggest a reflection on areas for improvement in the practice guidelines for mental health care in postpartum care.

## Figures and Tables

**Table 1:** Demographic characteristics of the sample.

	Women (n = 221–228) *	Men (n = 48–55) *
Age		
Age range (years)	18–48	25–49
(mean, SD)	(33.4; 2.8)	(35.3; 5.8)
Weeks pregnant		
Weeks pregnant: Range	4–40	12–40
(% 1st trimester; 2nd trimester; 3rd trimester)	(3.6%; 19.2%; 77.2%)	(2%; 22%; 76%)
Marital status		
Married/Partnered	94%	100%
Education level		
Tertiary (university)	43%	35%
Ethnicity		
Italian	81%	94%
Other European	13%	6%
Non-European	6%	0%
Other children		
No	61%	69%
Administration of measures		
Face-to-face	100%	2%
Telephone	0%	67%
At home	0%	31%
Reasons for hospitalization		
Risk of pre-term birth	20.17%	n.a.
General health reasons	22.80%	n.a.
Pregnancy complications	51.75%	n.a.
Data missing	5.26%	n.a.

* Numbers vary depending upon missing data across the questions.

**Table 2:** Women’s perceptions of the measures.

	Women (n = Varies per Question)
Both measures	
% Satisfied with each measure (n = 195–197)	
MGMQ	93%
Whooley/GAD-2	89%
Preference for the measures (n = 189)	
MGMQ preferred	50%
Whooley/GAD-2 preferred	37%
No preference	13%
MGMQ measure	
Q1 Response options: Preference (n = 146)	
Only include ‘Yes/No’	32%
Also include ‘Possibly’	67%
Unsure (n = 1)/Missing (n = 1)	1%
Q4 Response options: Preference (n = 146)	
Only include ‘Yes/No’	29%
Also include ‘Possibly’	71%
Q2 Impact Question (n = 123)	
Correct interpretation	100%
Whooley Measure	
Anhedonia Question comprehension (n = 144)	
Interpreted incorrectly	3% (n = 4)
Had difficulty understanding it	<1% (n = 1)
Attribution of endorsed Anhedonia Question (n = 17)	
Just due to physical changes	47% (n = 8)
Includes due to mood or worries	53% (n = 9)

**Table 3:** Percentage screening positive on the Whooley/GAD-2 and the MGMQ questions (n stated when 4 or less).

	Women (n = 227–228)	Men * (n = 53–55)
Measures: % screening positive		
Whooley/GAD-2 ^1^	35.5% (+/−5%)	20.0% (+/−8.8%)
Whooley ^2^	31.6% (+/−4.9%)	12.7% (+/−7.3%)
GAD-2 ^3^	15.8% (+/−3.8%)	10.9% (+/−6.9%)
Positive on both the Whooley&GAD-2	11.8% (+/−3.4%)	3.6% (n = 2)
MGMQ		
Minor or Mod/Major distress	33.9% (+/−5%)	15.1% (+/−7.9%)
Minor distress ^4^	7.0% (+/−2.7%)	1.9% (n = 1)
Moderate/Major distress ^5^	26.9% (+/−4.7%)	13.2% (+/−7.5%)
MGMQ Q2: % Bothered		
Moderately	19.8% (+/−4.2%)	11.3% (+/−7.0%)
A lot	4.0% (+/−2.1%)	1.9% (n = 1)
A little + ‘Possibly’ on Q4	7.0% (+/−2.7%)	1.9% (n = 1)
MGMQ Q4: % Wish-to-talk with a health professional		
Yes	7.5% (+/−2.8%)	0%
Possibly	16.2% (+/−3.9%)	3.8% (n = 2)
No	36.0% (+/−5.1%)	30.2% (+/−10.1%)
Not applicable ^a^	39.9% (+/−5.2%)	58.5% (+/−10.9%)
Missing:	n = 1	0%

Note: 90% Confidence intervals (CI) reported in brackets for the women’s data. For the men’s data, due to the small sample size, the 90% CI is only reported when the percentage is 10% or more. Otherwise, the n is reported. * Interpret these results with caution, due to the small sample size for men. ^1^ Positive on either, or both, of the Whooley or GAD-2. ^2^ At least one Whooley question answered ‘Yes’. ^3^ A total score across the 2 questions of 3 or more. ^4^ A little bit bothered (Q2) and ‘Possibly’ want to speak (Q4). ^5^ ‘Moderately’ or ‘a lot’ bothered (Q2); and/or ‘Yes’ wants to talk (Q4). ^a^ Responded ‘No’ on Q1, or ‘Not at all’ on Q2.

**Table 4:** Screen positive concordance between the Whooley/GAD-2 and the MGMQ.

Screening Outcomes (Positive/Negative)	Positive Whooley/GAD-2 ^1^ *f* (%)	Negative Whooley/GAD-2 *f* (%)	Total
Positive MGMQ ^2^	53 (68.8%)	24 (31,2%)	77 (33.77%)
Negative MGMQ	28 (18.54%)	123 (81.46%)	151 (66.23%)
Total	81 (35.52%)	147 (64.48%)	228

^1^ Positive on either, or both, of the Whooley or GAD-2. ^2^ Positive on the Minor or Moderate/Major distress classification (see text and Table 3).

## Data Availability

Data are available upon reasonable request to the authors.

## Abbreviations

The following abbreviations are used in this manuscript:

ASST	Azienda Socio Sanitaria Territoriale
DSM	Diagnostic and Statistical Manual of Mental Disorders
GAD-2	Generalized Anxiety Disorders-2
MGMQ	Matthey Generic Mood Questionnaire
NICE	National Institute for Health and Care Excellence
PHQ-2	Patient Health Questionnaire-2
PO	Project Officer
Q1 MGMQ	Question 1 of MGMQ, Distress Question
Q2 MGMQ	Question 2 of MGMQ, Impact Question (bother)
Q3 MGMQ	Question 3 of MGMQ, Reason Question
Q4 MGMQ	Question 4 of MGMQ, Wish-to-Talk Question
UK	United Kingdom
Whooley/GAD-2	Whooley questions used together with the GAD-2
